# Causal Effects of Overall and Abdominal Obesity on Insulin Resistance and the Risk of Type 2 Diabetes Mellitus: A Two-Sample Mendelian Randomization Study

**DOI:** 10.3389/fgene.2020.00603

**Published:** 2020-07-02

**Authors:** Hua Xu, Chuandi Jin, Qingbo Guan

**Affiliations:** ^1^Department of Endocrinology, Shandong Provincial Hospital Affiliated to Shandong University, Jinan, China; ^2^Shandong Clinical Medical Center of Endocrinology and Metabolism, Institute of Endocrinology and Metabolism, Shandong Academy of Clinical Medicine, Jinan, China; ^3^Institute for Medical Dataology, Shandong University, Jinan, China

**Keywords:** type 2 diabetes mellitus, insulin resistance, abdominal obesity, body fat mass, body fat mass distribution, Mendelian randomization

## Abstract

Overall and abdominal obesity were significantly associated with insulin resistance and type 2 diabetes mellitus (T2DM) risk in observational studies, though these associations cannot avoid the bias induced by confounding effects and reverse causation. This study aimed to test whether these associations are causal, and it compared the causal effects of overall and abdominal obesity on T2DM risk and glycemic traits by using a two-sample Mendelian randomization (MR) design. Based on summary-level statistics from genome-wide association studies, the instrumental variables for body mass index (BMI), waist-to-hip ratio (WHR), and WHR adjusted for BMI (WHRadjBMI) were extracted, and the horizontal pleiotropy was analyzed using MR–Egger regression and the MR–pleiotropy residual sum and outlier (PRESSO) method. Thereafter, by using the conventional MR method, the inverse-variance weighted method was applied to assess the causal effect of BMI, WHR, and WHRadjBMI on T2DM risk, Homeostatic model assessment of insulin resistance (HOMA-IR), fasting insulin, fasting glucose, and Hemoglobin A1c (HbA1c). A series of sensitivity analyses, including the multivariable MR (diastolic blood pressure, systolic blood pressure, high-density lipoprotein cholesterol, and low-density lipoprotein cholesterol as covariates), MR–Egger regression, weighted median, MR–PRESSO, and leave-one-out method, were conducted to test the robustness of the results from the conventional MR. Despite the existence of horizontal pleiotropy, consistent results were found in the conventional MR results and sensitivity analyses, except for the association between BMI and fasting glucose, and WHRadjBMI and fasting glucose. Each one standard deviation higher BMI was associated with an increased T2DM risk [odds ratio (OR): 2.741; 95% confidence interval (CI): 2.421–3.104], higher HbA1c [1.054; 1.04–1.068], fasting insulin [1.202; 1.173–1.231], and HOMA-IR [1.221; 1.187–1.255], similar to findings for causal effect of WHRadjBMI on T2DM risk [1.993; 1.704–2.33], HbA1c [1.061; 1.042–1.08], fasting insulin [1.102; 1.068–1.136], and HOMA-IR [1.127; 1.088–1.167]. Both BMI (*P* = 0.546) and WHRadjBMI (*P* = 0.443) were unassociated with fasting glucose in the multivariable MR analysis. In conclusion, overall and abdominal obesity have causal effects on T2DM risk and insulin resistance but no causal effect on fasting glucose. Individuals can substantially reduce their insulin resistance and T2DM risk through reduction of body fat mass and modification of body fat distribution.

## Introduction

Type 2 diabetes mellitus (T2DM) is a chronic metabolic disease characterized by hyperglycemia secondary to insulin resistance and pancreatic β-cell failure (Alejandro et al., 2015). The findings of human epidemiologic studies indicate that the global prevalence of T2DM is increasing rapidly, and this increase parallels the increase in the prevalence of obesity (Sampath Kumar et al., 2019). The body mass index (BMI) is routinely used to quantify the overall obesity although body fat distribution of individuals can vary substantially. The waist-to-hip ratio (WHR) and WHR adjusted for BMI (WHRadjBMI) are frequently used surrogate measures of abdominal obesity. Many observational epidemiologic studies, including case–control and cohort studies, have demonstrated that higher WHR and BMI are two important risk factors for developing T2DM (Vazquez et al., 2007; Lv et al., 2017). Moreover, cohort and cross-sectional studies (Wang et al., 2018; Benites-Zapata et al., 2019) demonstrated that BMI and WHR were associated with glycemic traits, including fasting insulin, Hemoglobin A1c (HbA1c), and insulin resistance [measured by Homeostatic model assessment of insulin resistance (HOMA-IR)]. Longitudinal and cross-sectional studies have found an association between increased risk of T2DM and higher genetic predisposition to both BMI and WHRadjBMI in European and East Asian populations (Robiou-du-Pont et al., 2013; Zhu et al., 2014; Huang et al., 2015).

However, these observational studies cannot avoid the bias induced by the confounding effect and reverse causation and, therefore, are incapable of confirming whether these associations are causal (Smith and Ebrahim, 2003). Mendelian randomization (MR) is an approach that is used to unbiasedly test or estimate the causal relationship between an exposure and an associated outcome by using data on inherited genetic variants that influence exposure status in the presence of unmeasured confounding (Didelez and Sheehan, 2007; Lawlor et al., 2008). In the past few years, MR has been extensively used in epidemiology and other related areas of population science (Smit et al., 2019; Wainberg et al., 2019; Yang et al., 2019).

Previous MR analyses have demonstrated that per 1 standard deviation (SD) higher WHRadjBMI and BMI were causally associated with T2DM risk in European populations (Dale et al., 2017; Emdin et al., 2017). Wang et al. (2018) conducted an MR to further investigate the causal effect of both BMI and WHR on glycemic traits, and they found that BMI had a causal relevance for insulin secretion, whereas neither WHR and BMI was causally associated with HOMA-IR in a conventional MR in a Chinese Han population. However, there is a dearth of MR studies for testing and comparing the causal effect of both overall and abdominal obesity on glycemic in the European population. Epidemiologic studies have found differences in T2DM epidemiologic characteristics between the Asian and European population wherein, in comparison with South Asians, Europeans have a lower T2DM risk, typically develop T2DM 5–10 years later, and have a slower disease progression (Gujral et al., 2013; Admiraal et al., 2014; Gupta and Misra, 2016; Meeks et al., 2016; Banerjee and Shah, 2018). Moreover, Europeans developed many metabolic abnormalities, including hyperglycemia and elevated triacylglycerol and low high-density lipoprotein cholesterol (HDL-C), at a higher BMI and age (Raji et al., 2001; Razak et al., 2007). Therefore, the estimation and comparison of the causal effects of overall and abdominal obesity on glycemic traits could provide insights into the obesity-related mechanism of T2DM.

In this study, a two-sample Mendelian randomization (TSMR) with a large sample size was conducted to determine whether a genetic predisposition to increased BMI, WHR, and WHRadjBMI was causally associated with T2DM and glycemic traits, including HOMA-IR, fasting insulin, fasting glucose, and HbA1c. The causal effects were further compared to identify differences in the effect of overall and abdominal obesity on T2DM development and glycemic traits.

## Materials and Methods

### Data Source

This study aimed to explore the causal effect of WHR, BMI, and WHRadjBMI on the risk of T2DM and glycemic traits (HOMA-IR, fasting insulin, fasting glucose, and HbA1c) in an European population, and used diastolic blood pressure (DBP), systolic blood pressure (SBP), HDL-C, and low-density lipoprotein cholesterol (LDL-C) as the covariates. The genome-wide association study summary statistics datasets used in this study were obtained from Zenodo^1^ for WHR (Censin et al., 2019), BMI (Censin et al., 2019), and WHRadjBMI (Censin et al., 2019); the Program in Complex Trait Genomics^2^ for T2DM (Xue et al., 2018); MAGIC Consortium^3^ for HOMA-IR (Dupuis et al., 2010), fasting glucose (Manning et al., 2012), fasting insulin (Manning et al., 2012), and HbA1c (Wheeler et al., 2017); the MRBase platform^4^ for HDL-C (Kettunen et al., 2016) and LDL-C (Kettunen et al., 2016); and the MRC-IEU Consortium^5^ for SBP and DBP. Detailed information of the summary statistics datasets are displayed in Table 1. We obtained the β-coefficients and standard errors for the per allele association of each single-nucleotide polymorphism (SNP) as well as all exposures and outcomes from these data sources.

**TABLE 1:** Summary statistics of data source.

Traits	Consortium	Data sources	No. of participants	No. of Variants	Population	Units in TSMR
WHR	ZENODO	Censin; PloS Genet; 2019	697,734	27,381,301	European	SD
BMI	ZENODO	Censin; PloS Genet; 2019	806,834	27,376,273	European	SD
WHRadjBMI	ZENODO	Censin; PloS Genet; 2019	694,649	27,375,636	European	SD
HDL-C	MRBase	Kettunen; Nat Commun; 2016	21,555	11,865,530	European	SD
LDL-C	MRBase	Kettunen; Nat Commun; 2016	21,559	11,871,461	European	SD
SBP	MRC-IEU	Ben Elsworth; 2018	436,419	9,851,867	European	SD
DBP	MRC-IEU	Ben Elsworth; 2018	436,424	9,851,867	European	SD
Fasting glucose	MAGIC	Manning, Nat Genet; 2012	58,074	2,628,880	European	mmol/L
Fasting insulin	MAGIC	Manning, Nat Genet; 2012	51,750	2,627,849	European	log pmol/L
HOMA-IR	MAGIC	Dupuis; Nat Genet; 2010	37,037	2,458,074	European	log HOMA
HbA1c	MAGIC	Wheeler, PloS Med; 2017	123,655	2,586,698	European	%
T2DM	Program in Complex Trait Genomics	Xue; Nat Commun; 2018	62,892/596,424	5,053,015	European	log odds

*WHR, waist-to-hip ratio; BMI, body mass index; WHRadjBMI, waist-to-hip ratio adjusted for body mass index; HDL-C, high-density lipoprotein cholesterol; LDL-C, low-density lipoprotein cholesterol; SBP, systolic blood pressure; DBP, diastolic blood pressure; T2DM, Type 2 diabetes; TSMR, two-sample Mendelian randomization; SD, standard deviation; HOMA-IR, Homeostasis model assessment of insulin resistance; HbA1c, Hemoglobin A1c.*

### Selection of Genetic Instrumental Variables

In the TSMR analysis conducted in this study, the genetic variants for exposures (BMI, WHR, and WHRadjBMI) were used as instrumental variables (IVs) and were obtained by two steps. Firstly, SNPs that are strongly associated with exposures (*P* < 5.0 × 10^–8^) were extracted. Secondly, we pruned these extracted SNPs by linkage disequilibrium (LD; *r*^2^ = 0.001, clumping distance = 10,000 kb) to ensure that each IV was independent of the others. To test the strength of the IVs, the *F*-statistics were calculated as previously described (Xu and Hao, 2017). *F*-statistics >10 are considered adequately strong to mitigate against any bias of the causal IV estimate.

### Heterogeneity and Horizontal Pleiotropic Analysis

In MR, heterogeneity in the causal estimate may indicate that a variant has an effect on the outcome outside of its effect on the exposure (known as horizontal pleiotropy), and this can cause severe bias (Davey Smith and Hemani, 2014). Mendelian randomization–Egger (MR–Egger) regression was undertaken to assess the horizontal pleiotropy of the IVs, where a regression intercept that significantly differed from zero (*P* < 0.05) indicated the presence of horizontal pleiotropy exists or that the InSIDE (INstrument Strength Independent of Direct Effect) assumption was violated (Bowden et al., 2015). Heterogeneity between IVs in the conventional MR, with the inverse-variance weighted (IVW) method, was estimated by *Cochran’s Q* statistic. The MR pleiotropy residual sum and outlier (MR–PRESSO) method can be used to test horizontal pleiotropic outliers and can obtain the corrected causal effect after removal of these outliers in MR (Verbanck et al., 2018). In the present study, both MR–Egger regression and MR–PRESSO tests were conducted using the TwoSampleMR and MRPRESSO R package in R (version 3.6.1), respectively.

### Mendelian Randomization

Mendelian randomization can test and estimate the causal effect of an exposure on an outcome by using genetic variants as the IVs (Zheng et al., 2017). Firstly, Wald ratios were calculated for each IV by dividing the per-allele log-odds ratio (or beta) of that variant in the outcome data by the log-odds ratio (or beta) of the same variant in the exposure data. Then, the random-effects IVW method was applied to estimate the association between exposures and outcomes. In IVW, the Wald ratio for each SNP was weighted by its inverse variance, and the effect estimates were meta-analyzed using random effects.

### Sensitivity Analysis

Sensitivity analysis was used to test the disproportionate effects of variants and the pleiotropy in the MR analysis (Mokry et al., 2016). A series of sensitivity analyses were conducted to test the robustness of the conventional MR results.

Multivariable IVW, which included the DBP, SBP, HDL-C, and LDL-C as covariates, was carried out in accordance with the method proposed by Rees et al. (2017) that was used to account for possible horizontal pleiotropy arising from the association of the instrument with these variables.

The MR–Egger regression and weighted median method are two pleiotropy-robust MR methods that are used to estimate consistent causal effects against unknown directional pleiotropy under the InSIDE assumptions (Bowden et al., 2015). In the MR–Egger regression method, the regression line fitted to the data is not constrained to pass through the origin, and the intercept represents the horizontal pleiotropic effect that may bias the IVW estimate, whereas the slope represents pleiotropy-corrected causal estimates. The weighted median method has considerable robustness to individual genetics with strongly outlying causal estimates and could provide a consistent causal estimate when the valid IVs exceed 50%.

The MR–PRESSO method was used to identify potential outliers in the conventional MR testing, and provided a robust estimate with outlier correction. Moreover, testing of significant distortion in the IVW causal estimate before and after MR–PRESSO correction, was undertaken and served as a sensitivity analysis.

The leave-one-out sensitivity analysis was conducted to ascertain whether the association was being disproportionately influenced by a single SNP. In this analysis, the random-effects IVW was repeated by leaving out each SNP in turn, and the overall analysis including all SNPs was used for the comparison. The variation of the results from before and after the removal of each SNP reflects the sensitivity of this SNP.

## Results

### Genetic IVs

A total of 546, 356, and 330 IVs were identified for BMI, WHR, and WHRadjBMI, respectively. Some IVs were absent in the outcome data; however, the *F* statistics for BMI-IVs (86.250–89.078), WHR-IVs (67.502–67.991), and WHRadjBMI-IVs (90.758–96.860) that were used for MR were more than 10, which indicated that the weak instrument bias was negligible. Detailed information of IVs used in this study are shown in Supplementary Table S1.

### Horizontal Pleiotropy and Heterogeneity Analysis

The MR–Egger regression intercepts obtained in this study (Table 2) showed that horizontal pleiotropy (*P* = 0.029) was only found in the MR with WHRadjBMI as exposure and fasting insulin as the outcome. Heterogeneity (Table 2) was observed between IVs; therefore, the random-effect IVW method was used in the subsequent stages of the research analysis. The MR–PRESSO test showed that horizontal pleiotropy was found in all IVW analyses in this study, and the horizontal pleiotropic outliers were identified and removed (Supplementary Table S2). After the removal of these outliers, the *F*-statistics of BMI-IVs (85.704–89.033), WHR-IVs (62.240–67.991), and WHRadjBMI-IVs (84.668–96.860) continued to remain well powered to estimate the causal effect of the exposure on the outcome.

**TABLE 2:** Heterogeneity and horizontal pleiotropy analysis.

Exposure	Outcome	Heterogeneity			Horizontal pleiotropy			
			
					MR–PRESSO test		MR–Egger regression	
						
		Q	Q df	*P*	Global Test RSSobs	Global Test *P*	intercepts (95% CI)	*P*
**BMI**								
	T2DM	2867.057	445	< 0.001	2885.029	< 0.001	−0.001 (0.994,1.005)	0.837
	HOMA-IR	510.260	446	0.019	512.445	0.023	0 (0.998,1.001)	0.430
	HbA1c	654.863	445	< 0.001	658.515	< 0.001	0 (0.999,1)	0.714
	Fasting insulin	624.170	448	< 0.001	626.889	< 0.001	0 (0.999,1.001)	0.842
	Fasting glucose	619.187	448	< 0.001	622.082	< 0.001	0 (0.999,1.001)	0.838
**WHR**								
	T2DM	1718.377	275	< 0.001	1739.466	< 0.001	0.008 (1,1.017)	0.055
	HOMA-IR	368.360	277	< 0.001	371.364	< 0.001	0 (0.998,1.002)	0.729
	HbA1c	413.286	279	< 0.001	416.611	< 0.001	0 (0.999,1.001)	0.695
	Fasting insulin	431.511	280	< 0.001	435.262	< 0.001	0 (0.998,1.002)	0.853
	Fasting glucose	358.324	280	0.001	361.045	0.001	0 (0.998,1.002)	0.935
**WHRadjBMI**								
	T2DM	1590.053	236	< 0.001	1609.124	< 0.001	0.003 (0.995,1.011)	0.455
	HOMA-IR	290.496	237	0.010	293.251	0.011	−0.001 (0.997,1.001)	0.175
	HbA1c	383.587	238	< 0.001	387.452	< 0.001	0 (0.999,1)	0.355
	Fasting insulin	362.009	239	< 0.001	365.491	< 0.001	−0.002 (0.997,1)	0.029
	Fasting glucose	276.448	239	0.048	278.957	0.047	−0.001 (0.998,1.001)	0.319

*WHR, waist-to-hip ratio; BMI, body mass index; WHRadjBMI, waist-to-hip ratio adjusted for body mass index; HDL-C, high-density lipoprotein cholesterol; LDL-C, low-density lipoprotein cholesterol; SBP, systolic blood pressure; DBP, diastolic blood pressure; T2DM, Type 2 diabetes; SD, standard deviation; HOMA-IR, Homeostasis model assessment of insulin resistance; HbA1c, Hemoglobin A1c; Q, *Cochran’s Q* test estimate; df, *Cochran’s Q* test degrees of freedom; MR–egger, Mendelian randomization–Egger regression; MR–PRESSO, Mendelian randomization pleiotropy residual sum and outlier method; OR, odds ratio; CI, confidence interval.*

### Causal Effect of WHR, BMI, and WHRadjBMI on T2DM and Glycemic Traits

**FIGURE 1:**
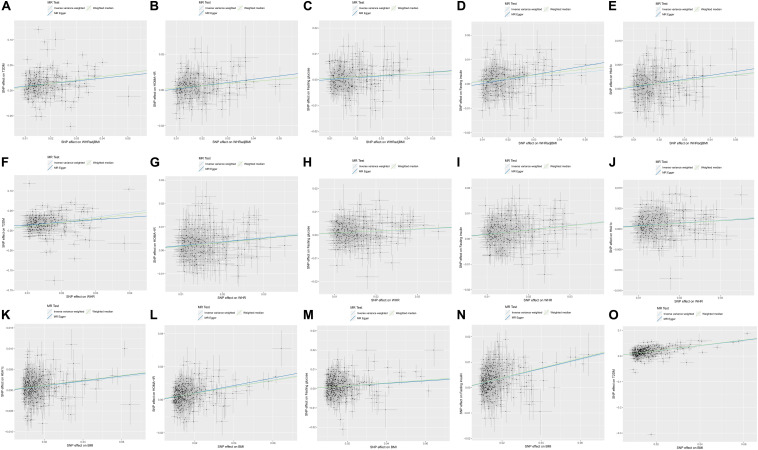
Scatterplot and causal effect of body mass index (BMI), waist-to-hip ratio (WHR), and WHR adjusted for BMI (WHRadjBMI) on type 2 diabetes mellitus (T2DM) and glycemic traits including HOMA-IR, fasting insulin, fasting glucose and glycate hemoglobin (HbA1c). **(A)** Causal effect of WHRadjBMI on T2DM, **(B)** Causal effect of WHRadjBMI on HOMA-IR, **(C)** Causal effect of WHRadjBMI on fasting glucose, **(D)** Causal effect of WHRadjBMI on fasting insulin, **(E)** Causal effect of WHRadjBMI on HbA1c, **(F)** Causal effect of WHR on T2DM, **(G)** Causal effect of WHR on HOMA-IR, **(H)** Causal effect of WHR on fasting glucose, **(I)** Causal effect of WHR on fasting insulin, **(J)** Causal effect of WHR on HbA1c, **(K)** Causal effect of BMI on T2DM, **(L)** Causal effect of BMI on HOMA-IR, **(M)** Causal effect of BMI on fasting glucose, **(N)** Causal effect of BMI on fasting insulin, and **(O)** Causal effect of BMI on HbA1c. The *x*-axis presents the single nucleotide polymorphism (SNP) effect on exposure, and the *y*-axis presents the SNP effect on outcome. The light blue, dark blue and green regression line represents the inverse-variance weighted (IVW), Mendelian randomization (MR)–Egger, and weighted median estimate, respectively.

Table 3 and Figure 1 show the causal effect estimates of WHR, BMI, and WHRadjBMI on T2DM and glycemic traits. The TSMR analysis by the IVW method showed a significant causal effect, wherein each SD of genetically higher BMI was associated with an increased T2DM risk [OR: 2.741; 95% confidence interval (CI): 2.421–3.104], higher fasting glucose [1.073; 1.048–1.099], higher fasting insulin [1.202; 1.173–1.231], higher HOMA-IR [1.221; 1.187–1.255], and higher HbA1c [1.054; 1.04–1.068]. Each SD of genetically higher WHR was associated with increased T2DM risk [3.12; 2.653–3.668], higher fasting glucose [1.087; 1.054–1.12], higher fasting insulin [1.193; 1.153–1.234], higher HOMA-IR [1.203; 1.155–1.252], and higher HbA1c [1.075; 1.056–1.095]. Each SD of genetically higher WHRadjBMI was associated with increased T2DM risk [1.993; 1.704–2.33], higher fasting glucose [1.039; 1.012–1.067], higher fasting insulin [1.102; 1.068–1.136], higher HOMA-IR [1.127; 1.088–1.167], and higher HbA1c [1.061; 1.042–1.08].

**TABLE 3:** Mendelian randomization results.

		BMI			WHR			WHRadjBMI		
				
Outcome	Method	nSNP	OR (95% CI)	*P*	nSNP	OR (95% CI)	*P*	nSNP	OR (95% CI)	*P*
**T2DM**										
	IVW	446	2.741(2.421,3.104)	6.12E-57	276	3.12(2.653,3.668)	3.87E-43	237	1.993(1.704,2.33)	5.59E-18
	MR–PRESSO (Outlier-corrected)	433	3.064(2.871,3.271)	9.15E-123	254	3.543(3.164,3.968)	2.67E-60	211	2.117(1.908,2.35)	3.06E-32
	Multivariable MR	842	3.338(2.625,4.246)	1.47E-20	672	2.887(2.54,3.281)	3.66E-50	633	2.105(1.755,2.524)	8.57E-15
	Weighted median	446	2.835(2.576,3.12)	6.18E-101	276	2.769(2.43,3.156)	1.02E-52	237	1.983(1.764,2.229)	1.74E-30
	MR–Egger	446	2.829(2.042,3.918)	9.28E-10	276	1.894(1.109,3.234)	0.020	237	1.699(1.087,2.656)	0.0201
**HOMA-IR**										
	IVW	447	1.221(1.187,1.255)	6.69E-44	278	1.203(1.155,1.252)	4.03E-19	238	1.127(1.088,1.167)	3.69E-11
	MR–PRESSO (Outlier-corrected)	446	1.223(1.19,1.258)	5.15E-38	278	NA	NA	238	NA	NA
	Multivariable MR	723	1.294(1.187,1.411)	1.29E-08	554	1.192(1.148,1.237)	1.28E-18	529	1.197(1.134,1.262)	1.61E-10
	Weighted median	447	1.214(1.161,1.27)	2.79E-17	278	1.18(1.114,1.251)	2.25E-08	238	1.128(1.07,1.188)	6.90E-06
	MR–Egger	447	1.255(1.165,1.352)	4.41E-09	278	1.175(1.024,1.348)	0.022	238	1.199(1.089,1.32)	2.72E-3
**HbA1c**										
	IVW	446	1.054(1.04,1.068)	4.99E-14	280	1.075(1.056,1.095)	1.13E-14	239	1.061(1.042,1.08)	3.85E-11
	MR–PRESSO (Outlier-corrected)	441	1.05(1.036,1.064)	9.67E-13	278	1.075(1.056,1.094)	4.41E-14	235	1.054(1.037,1.072)	1.61E-09
	Multivariable MR	735	1.076(1.031,1.123)	8.11E-3	569	1.074(1.055,1.093)	5.04E-14	543	1.065(1.038,1.094)	3.79E-06
	Weighted median	446	1.06(1.04,1.081)	3.83E-09	280	1.07(1.044,1.096)	4.60E-08	239	1.059(1.034,1.085)	2.08E-06
	MR–Egger	446	1.06(1.024,1.099)	0.001	280	1.062(0.998,1.131)	0.058	239	1.084(1.033,1.137)	0.001
**Fasting insulin**										
	IVW	449	1.202(1.173,1.231)	4.73E-50	281	1.193(1.153,1.234)	2.28E-24	240	1.102(1.068,1.136)	6.37E-10
	MR–PRESSO (Outlier-corrected)	446	1.198(1.17,1.226)	4.55E-42	280	1.201(1.162,1.241)	3.45E-23	237	1.108(1.077,1.141)	2.80E-11
	Multivariable MR	742	1.298(1.204,1.4)	6.62E-11	574	1.184(1.147,1.223)	5.81E-23	548	1.179(1.125,1.235)	2.18E-11
	Weighted median	449	1.213(1.168,1.26)	1.54E-23	281	1.187(1.136,1.24)	1.41E-14	240	1.129(1.083,1.177)	1.03E-08
	MR–Egger	449	1.209(1.134,1.289)	1.06E-08	281	1.18(1.052,1.324)	0.005	240	1.2(1.105,1.304)	2.29E-05
**Fasting glucose**										
	IVW	449	1.073(1.048,1.099)	4.53E-09	281	1.087(1.054,1.12)	8.78E-08	240	1.039(1.012,1.067)	0.004
	MR–PRESSO (Outlier-corrected)	446	1.083(1.059,1.107)	7.61E-12	278	1.098(1.067,1.13)	7.19E-10	239	1.044(1.017,1.072)	0.001
	Multivariable MR	742	1.025(0.947,1.109)	0.546	574	1.067(1.034,1.102)	8.18E-05	548	1.019(0.971,1.069)	0.443
	Weighted median	449	1.077(1.041,1.114)	1.88E-05	281	1.092(1.048,1.138)	2.60E-05	240	1.058(1.017,1.101)	0.005
	MR–Egger	449	1.067(1.002,1.136)	0.043	281	1.091(0.984,1.209)	0.098	240	1.075(1.001,1.155)	0.049

*WHR, waist-to-hip ratio; BMI, body mass index; WHRadjBMI, waist-to-hip ratio adjusted for body mass index; HDL-C, high-density lipoprotein cholesterol; LDL-C, low-density lipoprotein cholesterol; SBP, systolic blood pressure; DBP, diastolic blood pressure; T2DM, type 2 diabetes; SD, standard deviation; HOMA-IR, homeostasis model assessment of insulin resistance; HbA1c, hemoglobin A1c; nSNP, numbers of the SNPs (instrumental variable) used in Mendelian randomization; IVW, inverse-variance weighted; MR–egger, Mendelian randomization–Egger regression; MR–PRESSO, Mendelian randomization pleiotropy residual sum and outlier method; OR, odds ratio; CI, confidence interval.*

### Sensitivity Analysis

In the leave-one-out sensitivity analysis, no single SNP strongly or reversely drove the overall effect of exposure on outcome in the IVW (Supplementary Figure S1). Consistent results were observed in the IVW after the MR–PRESSO correction, MR–Egger regression, and the weighted median method, with the exception of the causal estimates of WHR on HbA1c (*P* = 0.058) and fasting glucose (*P* = 0.098) in the MR–Egger regression. The MR–Egger regression could obtain pleiotropy-corrected causal estimates, although this method had less statistical power than an equivalent IVW method, and the CIs were wider and included the null value (Bowden, 2017; Weng et al., 2018). Because the intercept of the MR–Egger regression indicates that there was no horizontal pleiotropy in the MR–Egger regression between WHR and both HbA1c (*P* = 0.695) and fasting glucose (*P* = 0.935), the causal estimate was more convincing in the IVW. In the multivariable IVW (DBP, SBP, HDL-C, and LDL-C as covariates), BMI (*P* = 0.546) and WHRadjBMI (*P* = 0.443) were not causally associated with fasting glucose, whereas other multivariable IVW results persisted with that in the univariable IVW.

Taken together, the causal effect estimates of BMI and WHRadjBMI on fasting glucose in conventional MR might be biased by the horizontal pleiotropy of SBP, DBP, HDL-C, and LDL-C, while no significant bias was found in other causal effect estimates despite the existence of horizontal pleiotropy and heterogeneity.

## Discussion

Numerous observational studies indicated that obesity was strongly associated with T2DM risk and glycemic traits (Lv et al., 2017), however, a causal effect cannot be ascertained from these studies due to residual confounding or reverse causality. This present study utilized a TSMR design that was applied to the summary-level data from a large-scale genome-wide association study to address the potential causal role of overall obesity (measured by BMI) and abdominal obesity (measure by WHRadjBMI) on the risk of T2DM and glycemic traits. The well-powered conventional MR (random-effect IVW method) confirmed that genetic predisposition to higher BMI, WHR, and WHRadjBMI are causally associated with higher fasting glucose, fasting insulin, HOMA-IR, HbA1c, and increased risk of T2DM in the European population.

However, heterogeneity and horizontal pleiotropy was found in the conventional MR analysis, a series of sensitivity analyses that included the multivariable MR (DBP, SBP, HDL-C, and LDL-C as covariates), MR–Egger regression, weighted median method, MR–PRESSO method, and leave-one-out analysis to test the robustness of the conventional MR results. The causal effect of BMI and WHRadjBMI on T2DM risk, HbA1c, fasting insulin, and HOMA-IR in the conventional MR were consistent with that in all the sensitivity analyses, which suggested that the causal estimate was robust and unbiased.

Each SD of genetically higher BMI [2.741; 2.421–3.104] and WHRadjBMI [1.993; 1.704–2.33] was associated with increased T2DM risk. Human epidemiologic studies have considered obesity to be a major risk factor of T2DM, and the substantial increase in the incidence of obesity contributes to the current T2DM epidemic (Sampath Kumar et al., 2019). Using the MR method in the European descendants, Emdin et al. confirmed that a 1 SD genetic increase in WHRadjBMI was associated with a higher risk of T2DM [1.77; 1.57–2.00] (Emdin et al., 2017), and Dale et al. revealed that each SD higher BMI was associated with increased T2DM risk [1.98; 1.41–2.78] (Dale et al., 2017). The results of this study are in agreement with those of previous observational studies (Lv et al., 2017; Sampath Kumar et al., 2019) and MR studies (Dale et al., 2017; Emdin et al., 2017) which suggested that both overall and abdominal obesity play a causal role on T2DM risk in the European population. In addition, our MR studies suggested that the causal effect of overall obesity on T2DM risk was greater than that of abdominal obesity. Moreover, both BMI [1.054; 1.04–1.068] and WHRadjBMI [1.061; 1.042–1.08] were found to have a causal effect on HbA1c, which suggested that overall and abdominal obesity have a similar but small causal effect on the HbA1c.

Insulin resistance refers to a decreased physiological response of peripheral tissues to insulin action, which implies an impaired effect of insulin in lowering the blood glucose (Gelaye et al., 2010). This serves as the key mechanism and a major global driver of the T2DM condition (Roglic, 2016; Czech, 2017). The accumulation of body fat and abdominal body fat are risk factors for increased insulin resistance (Kohrt et al., 1993; Gobato et al., 2014), and high BMI and WHR were found to be positively correlated with insulin resistance in observational epidemiological studies (Gobato et al., 2014; Benites-Zapata et al., 2019; Lin et al., 2019). Wang et al. reported that higher BMI was causally correlated with increased Stumvoll first- and second-phase insulin secretion and HOMA-IR, whereas no causal relationship between WHR and HOMA-IR was found in a conventional MR study in the Chinese Han population (Wang et al., 2018). In Europeans, a previous MR study found that higher WHRadjBMI was causally associated with higher fasting insulin levels (Emdin et al., 2017). The present MR study provides a similar conclusion with regard to the European population, each SD of genetically higher WHRadjBMI [1.102; 1.068–1.136] and BMI [1.202; 1.173–1.231] was found to play a positive causal effect on higher fasting insulin. Furthermore, each SD of genetically higher BMI [1.221; 1.187–1.255] and WHRadjBMI [1.127; 1.088–1.167] was causally associated with the HOMA-IR. These results suggested that higher overall and abdominal obesity serve as causal risk factors of fasting insulin and insulin resistance in the European population. The findings of the present study are supported by experimental studies as well. Obesity could stimulate the formation of lipid metabolites, hormones, and cytokines, which involves changes in the insulin signaling pathway and the accelerated progression of insulin resistance (Patel and Abate, 2013; Balsan et al., 2015). Moreover, the causal effect of overall obesity on fasting insulin and insulin resistance is slightly greater than that of abdominal obesity. Thus, we highlighted that both mass and distribution of body fat play a causal role on insulin resistance and T2DM risk. This indicates that the development of therapies to modify the mass and distribution of body fat to reduce overall and abdominal obesity might contribute to the prevention and alleviation of T2DM and insulin resistance-related diseases.

Furthermore, although higher BMI and WHRadjBMI was found to be causally associated with higher fasting glucose in our conventional MR in the European population, no statistical significance was found between BMI and fasting glucose (*P* = 0.546) or with WHRadjBMI and fasting glucose (*P* = 0.443) in the multivariable MR (DBP, SBP, HDL-C, and LDL-C as covariates). The casual estimates of BMI and WHRadjBMI on fasting glucose in conventional MR might be biased by the horizontal pleiotropy of DBP, SBP, HDL-C, and LDL-C. These negative results warrant further investigation.

Through a comparison of the causal estimates of BMI and WHRadjBMI on glycemic traits (fasting glucose, fasting insulin, HOMA-IR, and HbA1c), this study further emphasizes that overall and abdominal obesity might increase the T2DM risk mainly via elevation of insulin resistance.

In conclusion, overall and abdominal obesity have a causal effect on the T2DM risk and insulin resistance, and overall obesity may have stronger effects, whereas they may have no causal effect on the fasting glucose. These results suggest that individuals can substantially reduce their insulin resistance and T2DM risk through reduction of body fat mass and modification of body fat distribution.

## Data Availability Statement

Publicly available datasets were analyzed in this study. This data can be found here: The Zenodo (https://zenodo.org), the Program in Complex Trait Genomics (https://cnsgenomics.com), the MAGIC Consortium (http://www.magicinvestigators.org/), the MRBase platform (http://www.mrbase.org), and the MRC-IEU Consortium (http://www.bristol.ac.uk/integrative-epidemiology/).

## Author Contributions

HX and CJ collected, analyzed, and interpreted the data. All authors conceived and designed the project and wrote and approved the final version fo the manuscript.

## Conflict of Interest

The authors declare that the research was conducted in the absence of any commercial or financial relationships that could be construed as a potential conflict of interest.

## Footnotes

1 https://zenodo.org

2 https://cnsgenomics.com

3 http://www.magicinvestigators.org/

4 http://www.mrbase.org

5 http://www.bristol.ac.uk/integrative-epidemiology/

## Supplementary Material

FIGURE S1 Leave-one-out sensitive analysis.
