# Supplementary figures and images for: Causal Effects of Overall and Abdominal Obesity on Insulin Resistance and the Risk of Type 2 Diabetes Mellitus: A Two-Sample Mendelian Randomization Study
Source: Front Genet. 2020 Jul 2;11:603. doi: 10.3389/fgene.2020.00603 (PMC7343715; doi:10.3389/fgene.2020.00603)

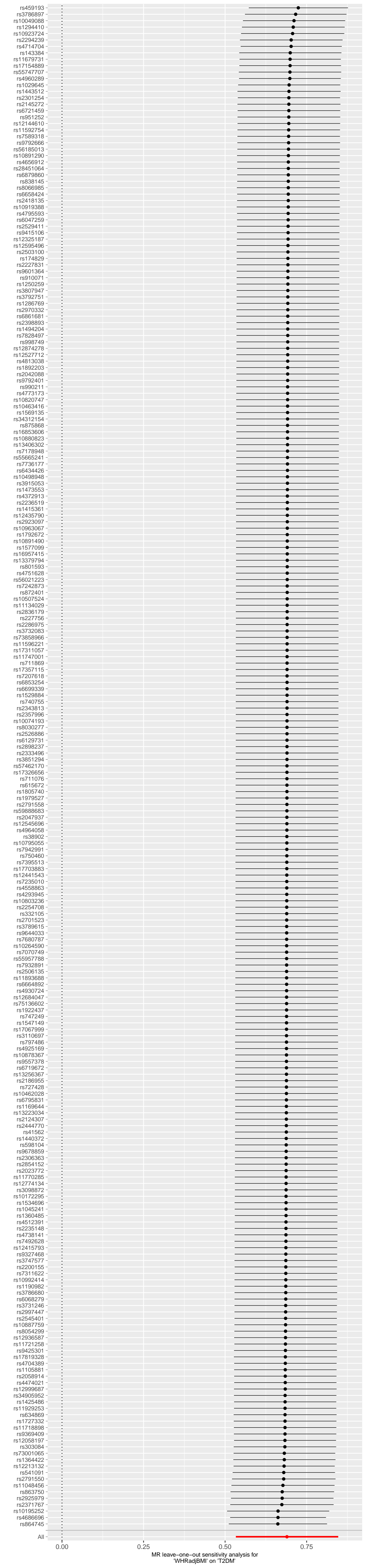

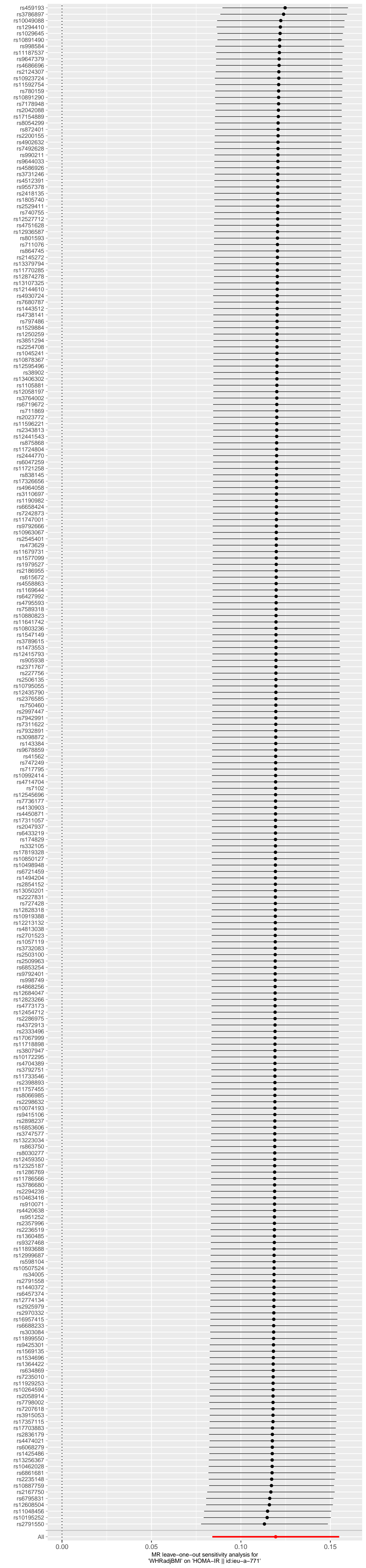

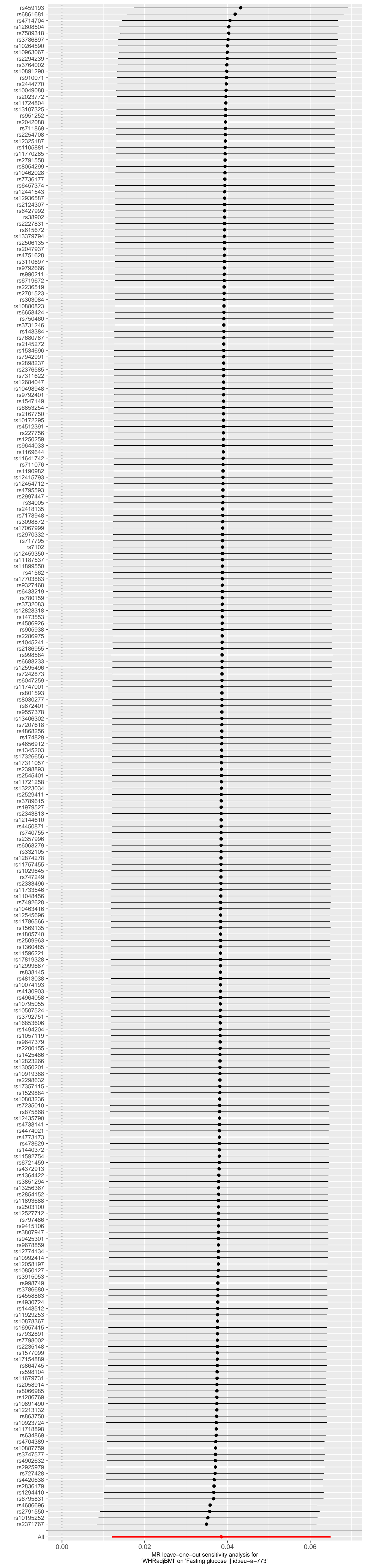

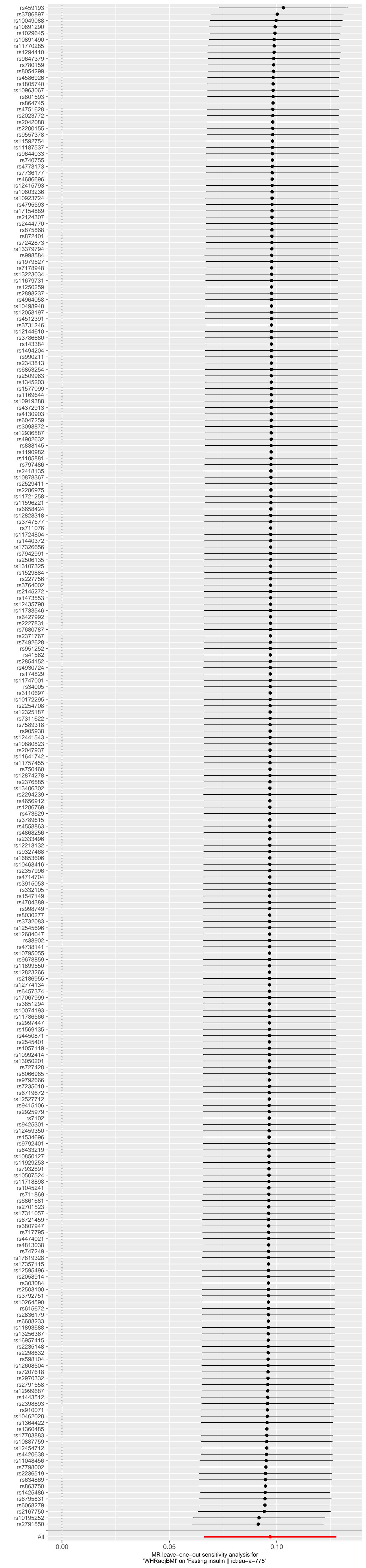

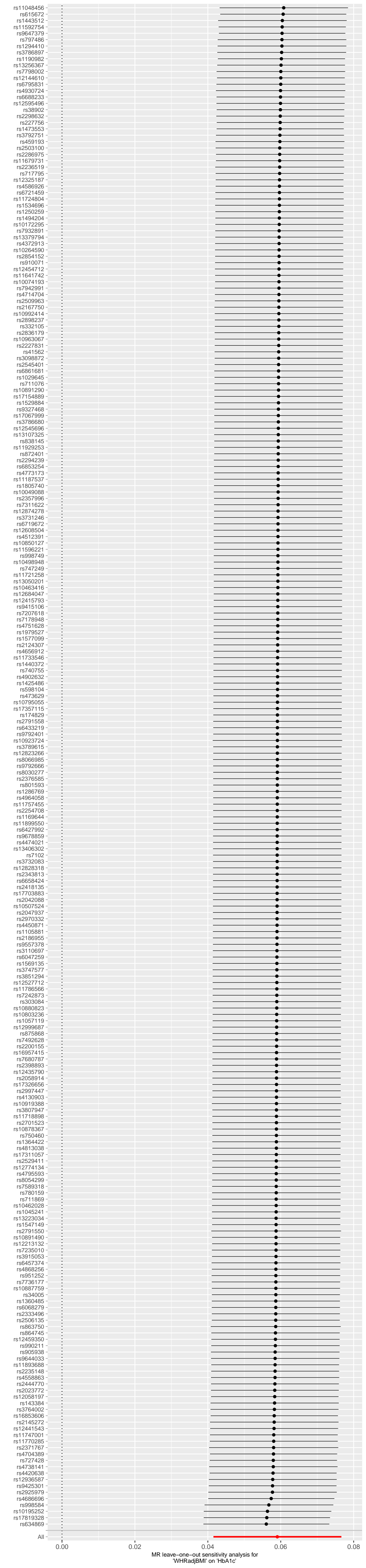

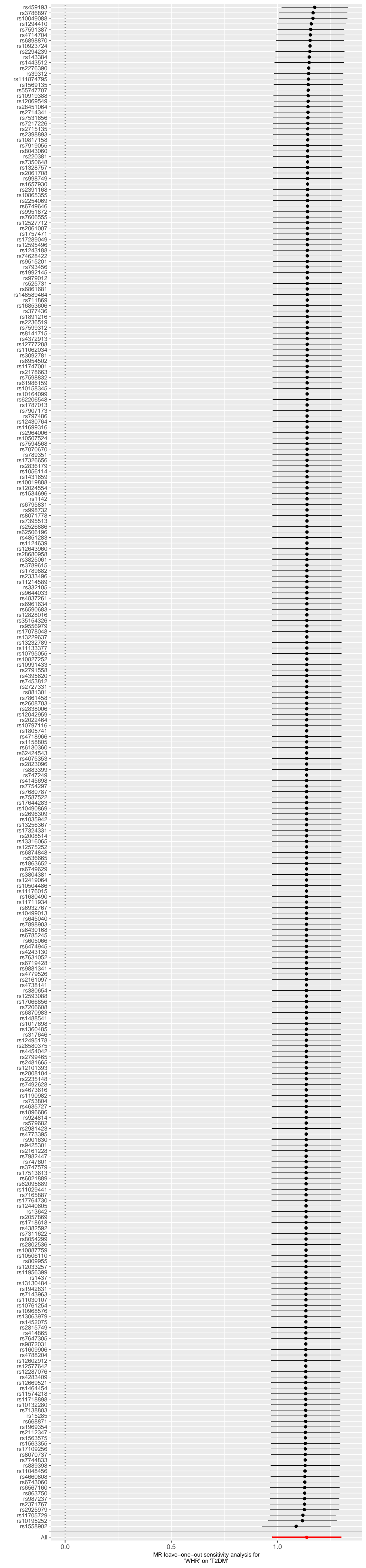

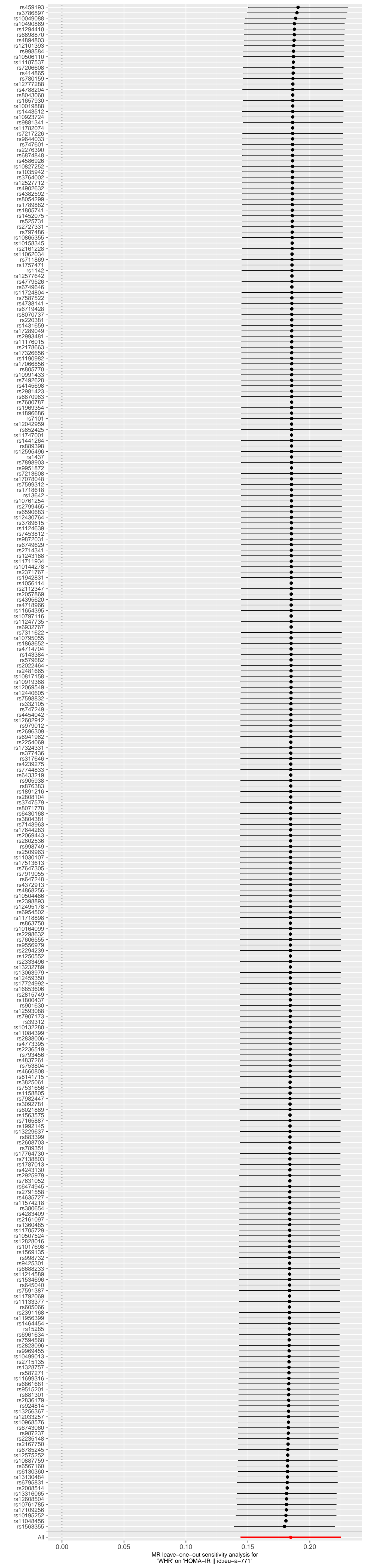

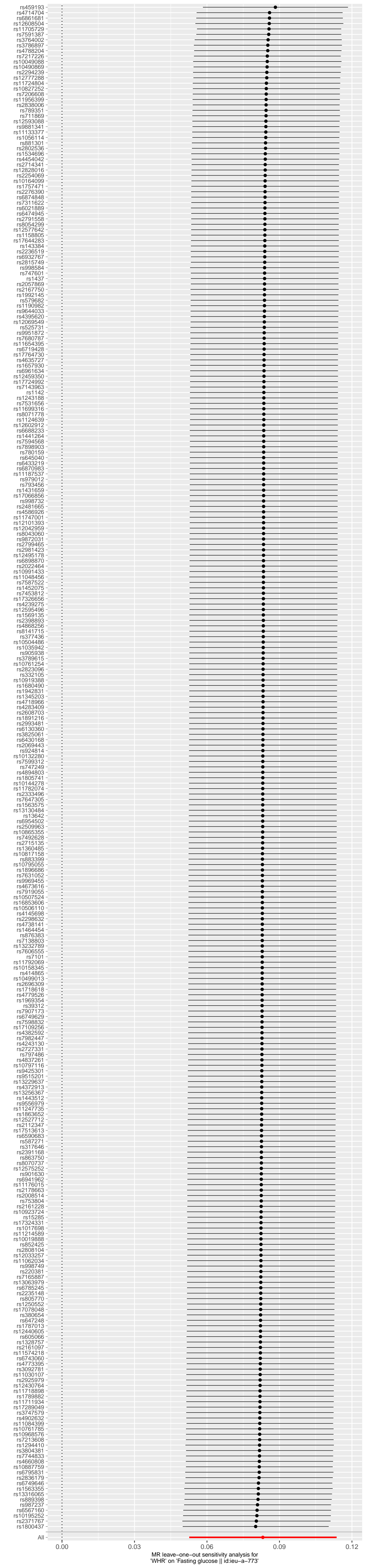

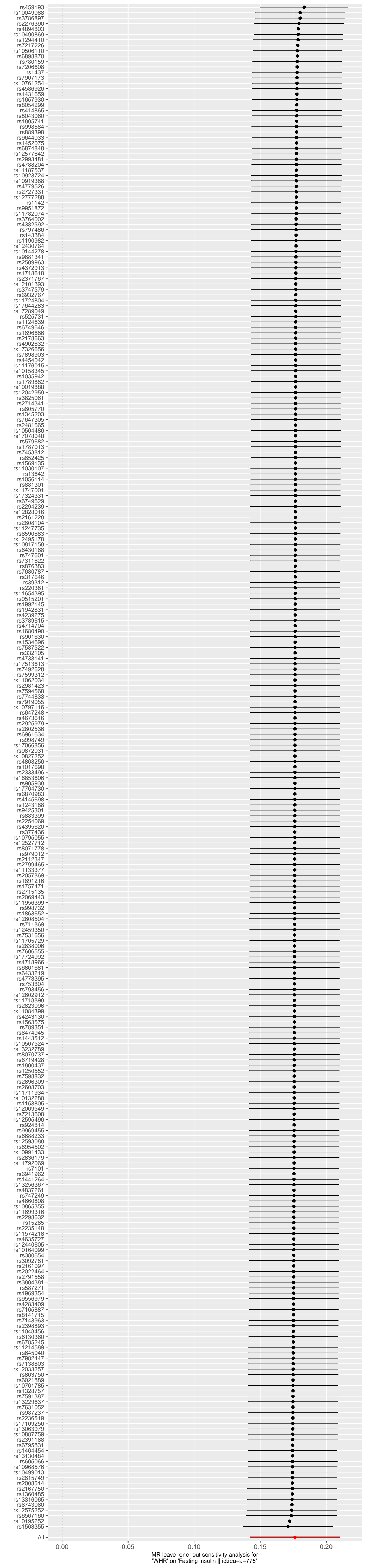

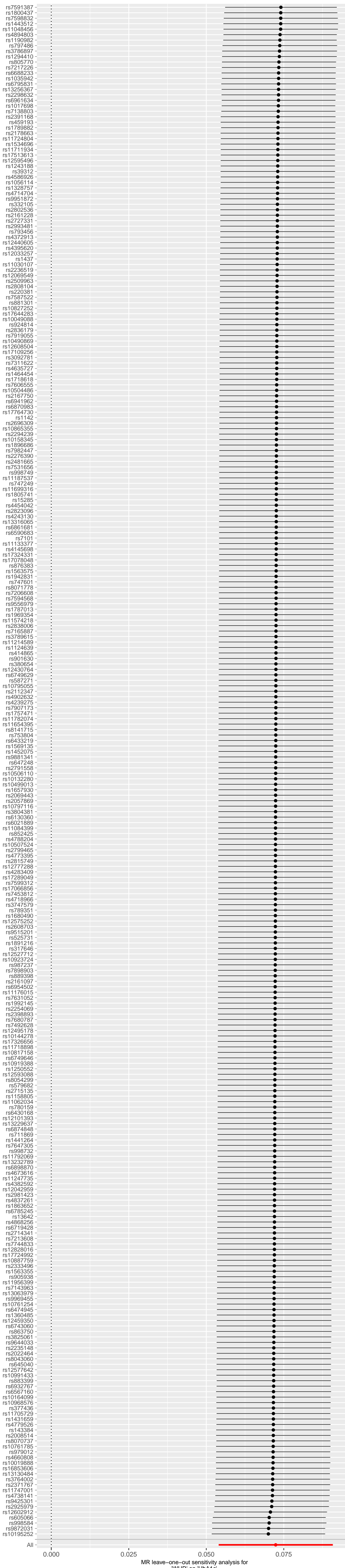

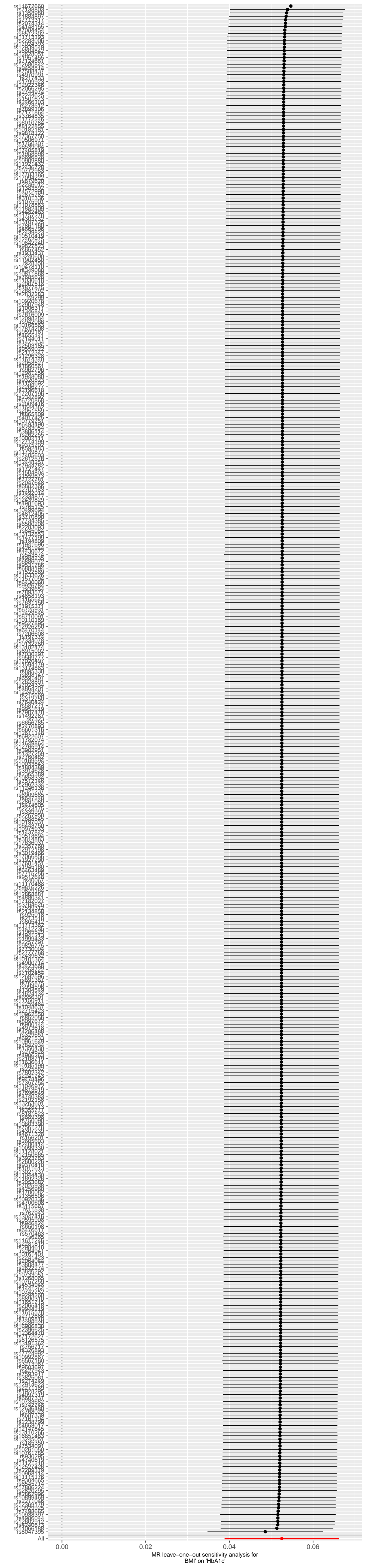

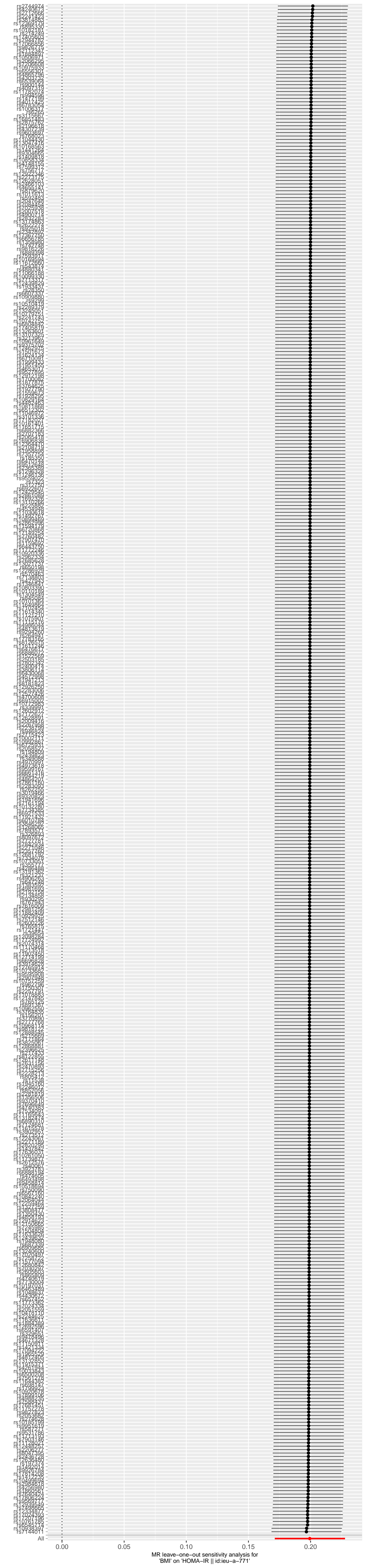

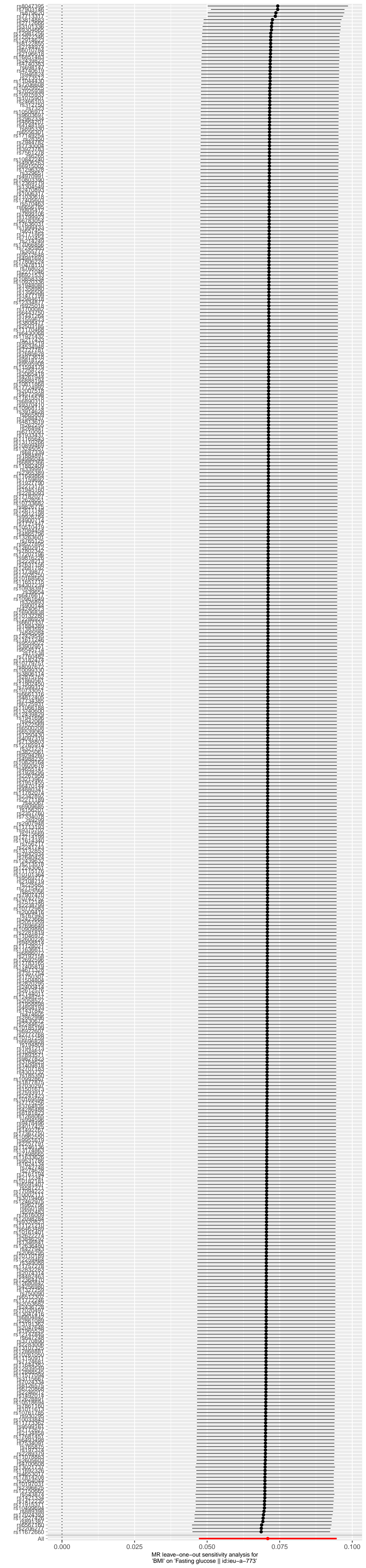

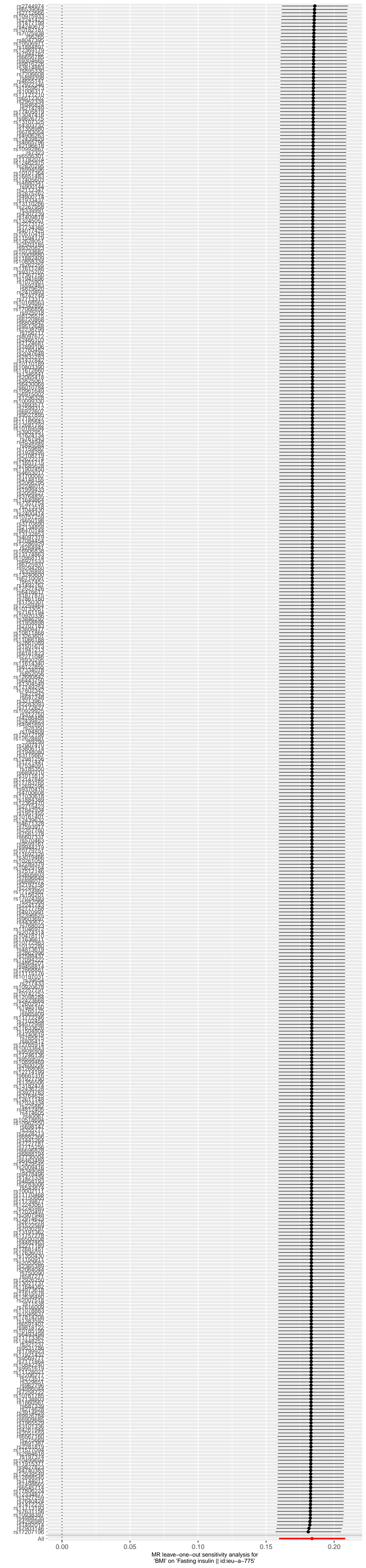

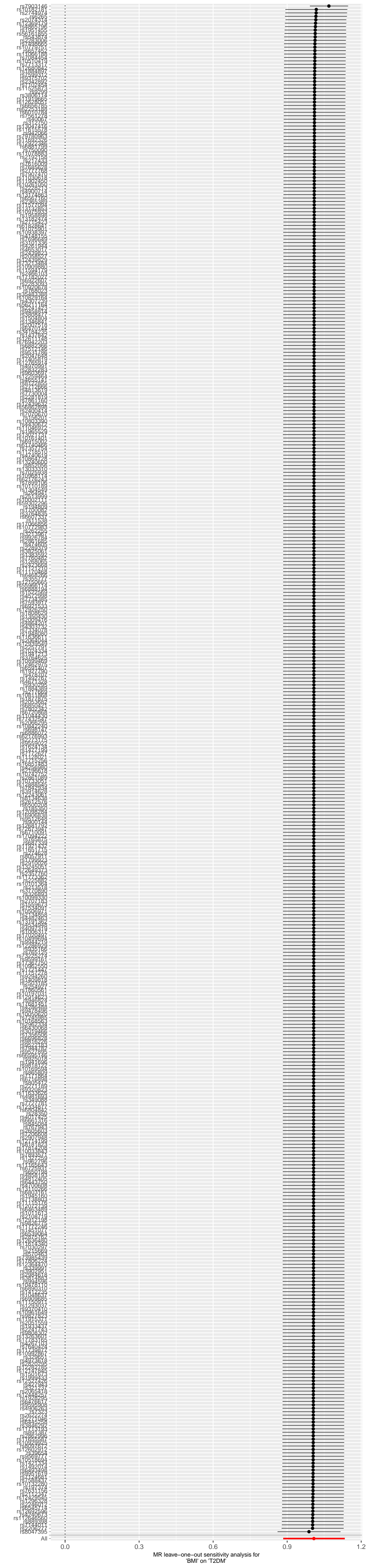

Supplement: FIGURE S1 — Leave-one-out sensitive analysis. [file Image_1.PDF]
